# Attitude Determination Using a MEMS-Based Flight Information Measurement Unit

**DOI:** 10.3390/s120100001

**Published:** 2011-12-22

**Authors:** Der-Ming Ma, Jaw-Kuen Shiau, I.-Chiang Wang, Yu-Heng Lin

**Affiliations:** Department of Aerospace Engineering, Tamkang University, Tamsui, New Taipei City 25137, Taiwan; E-Mails: derming@mail.tku.edu.tw (D.-M.M.); ichiangwang@gmail.com (I.-C.W.); jw93335@hotmail.com (Y.-H.L.)

**Keywords:** attitude determination, quaternion, flight information measurement unit, extended Kalman filter

## Abstract

Obtaining precise attitude information is essential for aircraft navigation and control. This paper presents the results of the attitude determination using an in-house designed low-cost MEMS-based flight information measurement unit. This study proposes a quaternion-based extended Kalman filter to integrate the traditional quaternion and gravitational force decomposition methods for attitude determination algorithm. The proposed extended Kalman filter utilizes the evolution of the four elements in the quaternion method for attitude determination as the dynamic model, with the four elements as the states of the filter. The attitude angles obtained from the gravity computations and from the electronic magnetic sensors are regarded as the measurement of the filter. The immeasurable gravity accelerations are deduced from the outputs of the three axes accelerometers, the relative accelerations, and the accelerations due to body rotation. The constraint of the four elements of the quaternion method is treated as a perfect measurement and is integrated into the filter computation. Approximations of the time-varying noise variances of the measured signals are discussed and presented with details through Taylor series expansions. The algorithm is intuitive, easy to implement, and reliable for long-term high dynamic maneuvers. Moreover, a set of flight test data is utilized to demonstrate the success and practicality of the proposed algorithm and the filter design.

## 1. Introduction

Obtaining an accurate vehicle attitude is essential for airplane navigation and control applications. The effectiveness of navigation and control is determined by the degree of precision of the navigation and control systems, including inertial measurement units. Traditional units—such as gimbaled gyroscopes, laser gyroscopes, and fiber optic gyroscopes and accelerometers—provide high-precision information for navigational calculations [1]; they are, however, expensive and bulky. With the maturation and advancement of semiconductor manufacturing technology, MEMS sensors are increasingly used in flight attitude calculations [2–9].

The attitude of the aero-vehicle can be determined by integrating the angular rates (pitch, roll, and yaw rates) of the vehicle. Nevertheless, accuracy requirements usually cannot be satisfied by using the inexpensive MEMS sensors. Therefore, some forms of Kalman filtering or complementary fusion algorithms are normally employed to provide more accurate and reliable attitude angles in the MEMS attitude determination systems. The pitch and roll angles can be estimated by using the gravity components from the measurement of the accelerometers [2]. Gravity acceleration cannot be determined accurately from the accelerometer measurements, especially in long-term high dynamic maneuvers. To increase the accuracy and reliability of the attitude determination, a considerable number of advanced fusion technologies using MEMS sensors were proposed in the literature. Using a triad of rate gyros fused with an ultra-short baseline, the differential GPS attitude determination system was presented in [2]. In [4], a gyro-free attitude determination was discussed by using accelerometers, magnetic sensors, and GPS as the primary sensors. A fuzzy logic based closed-loop strapdown attitude system for UAV is presented in [5]. In [7], a constrained Kalman filter was developed to eliminate the effects of kinetic accelerations in the y and z axes in the body frame, while using a differential speed signal to correct the kinetic acceleration of the x axis. A gain-scheduled complementary filter augmented by an acceleration-based switching architecture was proposed in [9] to provide robust performance, even when the vehicle is subject to strong acceleration. A miniature MEMS-based attitude and heading reference system, which makes use of an extended Kalman filter with adaptive gain, and with accelerometers and magnetic sensor as the measurements, was presented in [10]. A precision attitude determination employing a multiple model adaptive estimation scheme was reported in [11]. A Linear Fusion Algorithm for Attitude Determination Using Low Cost MEMS-Based Sensors is considered in [12]. In [13], an integrated MEMS INS/GPS and intelligent artificial neural network with Rauch-Tung-Striebel smoother scheme was proposed for position and orientation determination.

This study considers the utilization of a low-cost MEMS-based flight information measurement unit to determine attitude. The main contribution of this paper is that an attitude estimation algorithm is proposed using MEMs sensors. In the proposed algorithm, an extended Kalman filter is used instead of the classical channel-wise complementary filters for attitude estimation. Specifically, the roll and pitch angles computed from the gravity force decomposition, and the heading angle determined from the electronic compass unit, are selected as the measurement of the Kalman filter. This is not commonly used in airplane attitude determination. The immeasurable gravity accelerations are deduced from the outputs of the three axes accelerometers, the relative accelerations, and the accelerations due to body rotation. The proposed attitude determination algorithm employed the evolution of the four elements of the quaternion method as the dynamical model for the Kalman filter. The constraint of the four elements of the quaternion method is thus treated as a perfect measurement and is integrated into the filter design. By adopting a coupled and time-varying approach, the proposed algorithm performs well under different dynamic maneuvers. The proposed attitude estimation algorithm is intuitive and easy to implement, and due to the vehicle dynamics has been considered in the gravity computation, the algorithm can handle the situation of the long-term high dynamic maneuvers. Detailed derivations establish the approximations of the time varying noise variances of the measured signals.

The proposed algorithm is demonstrated through a set of flight test data collected from the in-house designed MEMS-based attitude determination system. The duration of the flight covers a total of 3,620 s. Details of the evolutions of the attitude angles are examined and explored in the paper. The results show the long-term stability and practicality of the proposed attitude estimation algorithm.

## 2. Flight Information Measurement Unit

**Figure 1.**
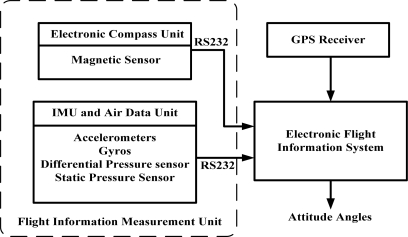
System Structure of the Flight Information Measurement Unit.

The determination of attitude considered in this study is based on a set of signals measured from an in house-designed Flight Information Measurement Unit (FIMU) [14] and a GPS receiver. The main function of the FIMU system is to measure the required signals for navigation computation and estimation; this includes the accelerations, angular rates, magnetic fields, differential pressures, static pressure, and temperature in a real-time and continuous manner. Hence, the receiver (an on-board computer) can use this information to calculate the vehicle’s attitude, heading, airspeed, altitude, angle of attack, and temperature information. The basic system structure is shown in Figure 1.

**Figure 2.**
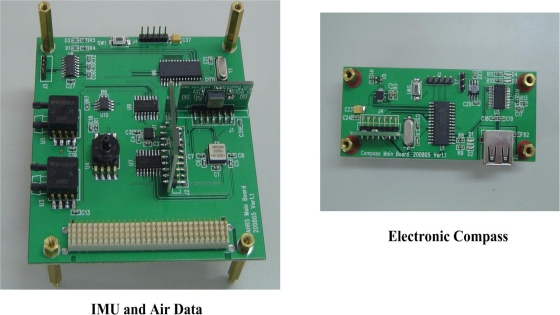
The Flight Information Measurement Unit.

To avoid or reduce electromagnetic interference during the measurement process (because the magnetic sensor is extremely sensitive to its ambient disturbances), the FIMU is split into two separate units, namely, an inertial and air data measurement unit, and an electronic compass unit. On the one hand, the inertial and air data measurement unit provides the capability for three-axis accelerations, three-axis angular rates, airspeed, altitude, temperature, and angle of attack measurements. The compass system, on the other hand, measures the magnitude of the external magnetic fields. A Microchip PIC micro-controller is used in each measuring unit to perform data acquisition and signal processing, and to transfer the results to the host system for further analysis and processing. An image of the FIMU is shown in Figure 2.

## 3. Attitude Determination

Determination of flight attitude involves the computation of aircraft pitch angle, roll angle, and yaw angle. Pitch angle and roll angle can be computed through the measured aircraft accelerations and body rates from the accelerometers and rate gyros. The heading angle is determined by computing the magnetic heading: a magnetic sensor measures the magnetic field, and corrects for the magnetic declination. With the measured three axes acceleration signals and pitch, roll, and yaw rate information—the pitch and roll angles can be determined either by computing the gravitational acceleration components on the body axes, or by using the Euler quaternion method [15]. Computation of the gravitational acceleration components provides long-term accuracy, although it is accompanied by high noise contents. The quaternion method, however, provides low noise contents and fast response to changes in the input signals, but tends to drift with time due to gyro bias errors. The results of pitch and roll computations (derived from gravitational acceleration components and the quaternion method) are mixed by means of complementary filtering, which is performed by implementing sensor data fusion techniques. This sensor data fusion technique can also be incorporated for heading computation by mixing the heading information from both the compass system and from the Euler quaternion method.

**Figure 3.**
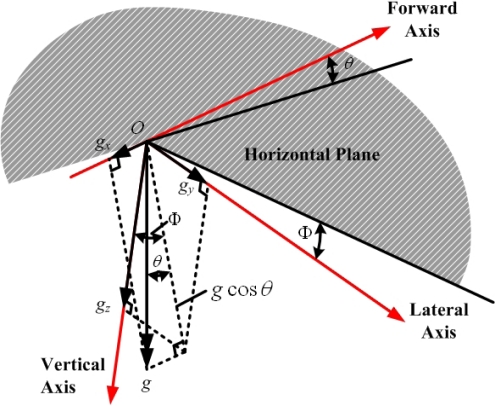
Gravitational force decomposition.

For example, assume that at a certain instant, the pitch angle of the aircraft is *θ*, roll angle is *ϕ*, and the gravitational force components along the body axes (X-, Y-, and Z-axis) are *g_x_*, *g_y_*, and *g_z_* respectively, as shown in Figure 3 [1]. The relationship between the gravitational acceleration components and the attitude angle are:

(1) $$\left[\begin{array}{c} g_{x}\\ g_{y}\\ g_{z} \end{array}\right]=\left[\begin{array}{c} -g\;\text{sin}\;\theta \\ g\;\text{sin}\;\phi \;\text{cos}\;\theta \\ g\;\text{cos}\;\phi \;\text{cos}\;\theta \end{array}\right]$$

Knowing *g_x_*, *g_y_*, and *g_z_*, the roll angle *ϕ* and pitch angle *θ* can be computed from:

(2) $$\phi ={\text{tan}}^{-1}\left(\frac{g_{y}}{g_{y}}\right),\;\;\;\theta ={\text{tan}}^{-1}\left(\frac{-g_{x}\;\text{cos}\;\phi }{g_{z}}\right)$$

**Figure 4.**
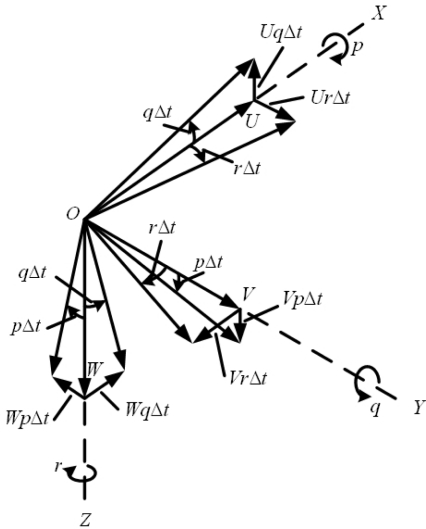
Vector change in velocity components due to angular rotation.

There are alternative ways to obtain *ϕ* and *θ* from Equation (1). They are mathematically equivalent. Computation of the gravity vector $g=\sqrt{g_{x}^{2}+g_{y}^{2}+g_{z}^{2}}$ is avoided by using Equation (2). But *g_x_*, *g_y_*, and *g_z_* cannot be measured directly during flight. The accelerations measured from the accelerometers are the total accelerations along the X-, Y-, and Z-axis (*a_x_*,*a_y_*,*a_z_*). The relationship between the measured accelerations (*a_x_*,*a_y_*,*a_z_*) and the gravitational force components, as shown in Figure 4 [1], are:

(3) $$\begin{array}{l} a_{x}=\dot{U}+(\mathrm{Wq}-\mathrm{Vr})+g_{x}\\ a_{y}=\dot{V}+(\mathrm{Ur}-\mathrm{Wp})+g_{y}\\ a_{z}=\dot{W}+(\mathrm{Vp}-\mathrm{Uq})+g_{z} \end{array}$$

where (*U*,*V*,*W*) are the inertial velocities along the X-, Y-, and Z-axis, whereas (*p*,*q*,*r*) are the pitch rate, roll rate, and yaw rate, respectively. Note that the local level frame is taken as the inertial frame in the study. We may thus derive (*g_x_*,*g_y_*,*g_z_*) by measuring or computing (*U*,*V*,*W*), (*U̇*, *V̇*, *Ẇ*), (*p*,*q*,*r*), and (*a_x_*,*a_y_*,*a_z_*); consequently, these results can be used to determine the aircraft pitch angle *θ* and roll angle *ϕ*. The advantage of this method is that no integral operation is involved in the computation. Thus, the measurement error and gyro biases are not accumulated, thus avoiding divergence in attitude computation. It therefore provides the results with long-term accuracy. However, due to the nature of the MEMS accelerometers and gyros and the computation of (*U*,*V*,*W*) and (*U̇*, *V̇*, *Ẇ*), the results usually lead to high noise contents.

**Figure 5.**
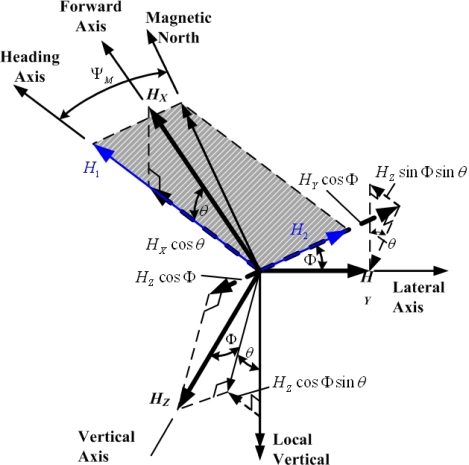
Magnetic force decomposition.

For magnetic heading computation, assume that the components of the Earth’s magnetic field along the X-, Y-, and Z-axis are *H_X_*, *H_Y_*, and *H_Z_*, respectively. Furthermore, the resolved components of *H_X_*, *H_Y_*, and *H_Z_* in the horizontal plane along the heading axis *H*_1_ and at right angles to the heading axis *H*_2_ (as shown in Figure 5) are given by [1]:

(4) $$H_{1}=H_{X}\;\text{cos}\;\theta +H_{Y}\;\text{sin}\;\phi \;\text{sin}\;\theta +H_{Z}\;\text{cos}\;\phi \;\text{sin}\;\theta$$

(5) $$H_{2}=H_{Y}\;\text{cos}\;\phi -H_{Z}\;\text{sin}\;\phi$$

Thus, the magnetic heading of the aircraft *ψ_M_* is:

(6) $$\psi_{M}={\text{tan}}^{-1}\;\frac{H_{2}}{H_{1}}$$

If the local magnetic declination is *ψ*_md_, the aircraft heading can be determined from *ψ* = *ψ_M_* + *ψ*_md_.

A common method used to compute the attitude angle is the Euler quaternion method [15], which uses the four symmetrical Euler parameters to define the aircraft attitude. The relationship between the attitude angles and the four parameters are:

(7) $$\left[\begin{array}{c} \phi \\ \theta \\ \psi \end{array}\right]=\left[\begin{array}{c} {\text{tan}}^{-1}(\frac{2(e_{0}e_{1}+e_{2}e_{3})}{e_{0}^{2}-e_{1}^{2}-e_{2}^{2}+e_{3}^{2}})\\ {\text{sin}}^{-1}2(e_{0}e_{3}-e_{1}e_{2})\\ {\text{tan}}^{-1}(\frac{2(e_{0}e_{3}+e_{1}e_{2})}{e_{0}^{2}+e_{1}^{2}-e_{2}^{2}+e_{3}^{2}}) \end{array}\right]$$

The four parameters are subjected to the following constraint equation:

(8) $$e_{0}^{2}+e_{1}^{2}+e_{2}^{2}+e_{3}^{2}=1$$

The dynamics of the four parameters regarding the aircraft body rate (*p*,*q*,*r*) are characterized in the following form:

(9) $$\left[\begin{array}{c} {\dot{e}}_{0}\\ {\dot{e}}_{1}\\ {\dot{e}}_{2}\\ {\dot{e}}_{3} \end{array}\right]=0.5\cdot \left[\begin{array}{cccc} 0 & -p & -q & -r\\ p & 0 & r & -q\\ q & -r & 0 & p\\ r & q & -p & 0 \end{array}\right]\;\left[\begin{array}{c} e_{0}\\ e_{1}\\ e_{2}\\ e_{3} \end{array}\right]$$

Therefore, the attitude angle in Equation (7) may be calculated by solving Equation (9). Only body information—(*p*,*q*,*r*) directly measured from rate gyros—is required to solve Equation (9). However, when MEMS gyros are used for body rate measurement, long-term drift is usually encountered due to gyro bias errors and integral operation for solving Equation (9).

## 4. Kalman Filter Design

As discussed in the previous section, the attitude computed from the gravitational force components provides long-term accuracy with high noise contents. The Euler quaternion method, however, provides less noisy results but suffers from a long-term drift problem. Either method alone may be inadequate for attitude computation. Hence, in this study, the extended Kalman filter [16] is implemented to integrate the attitude computation from the gravitational force components and from the Euler quaternion method.

The Kalman filter is a model-based estimation technique. The dynamics that characterize the relationship between the aircraft body rate (pitch, roll, and yaw rates) and the four parameters of the quaternion method described in Equation (9) comprises the dynamic model of the Kalman filter. The relationship between the attitude angle and the four parameters in Equation (7) is chosen as the output equation of the filter. Thus, the dynamic system can be expressed as:

(10) $$\begin{array}{l} \dot{x}=\mathrm{Ax}+w\\ y=h(x)+v \end{array}$$

where *x* = [*e*_0_ *e*_1_ *e*_2_ *e*_3_]*^T^* is the state of the system, *y* = [*ϕ θ* *ψ* 1]*^T^* is the output, and *w* and *v* are the precess and measurement noise, respectively. The system matrix *A* and output function *h*(*x*) are defined as:

(11) $$A=\left[\begin{array}{cccc} 0 & -\frac{p}{2} & -\frac{q}{2} & -\frac{r}{2}\\ \frac{p}{2} & 0 & \frac{r}{2} & -\frac{q}{2}\\ \frac{q}{2} & -\frac{r}{2} & 0 & \frac{p}{2}\\ \frac{r}{2} & \frac{q}{2} & -\frac{p}{2} & 0 \end{array}\right]\;\;\;;\;\;\;h(x)=\left[\begin{array}{c} {\text{tan}}^{-1}(\frac{2(e_{0}e_{1}+e_{2}e_{3})}{e_{0}^{2}-e_{1}^{2}-e_{2}^{2}+e_{3}^{2}})\\ {\text{sin}}^{-1}2(e_{0}e_{3}-e_{1}e_{2})\\ {\text{tan}}^{-1}(\frac{2(e_{0}e_{3}+e_{1}e_{2})}{e_{0}^{2}+e_{1}^{2}-e_{2}^{2}+e_{3}^{2}})\\ e_{0}^{2}+e_{1}^{2}+e_{2}^{2}+e_{3}^{2} \end{array}\right]$$

The measurements of pitch angle *θ* and roll angle *ϕ* are determined from the gravitation force components Equation (1), while the heading angle *ψ* is determined from the measured magnetic heading Equation (6), and is corrected with local magnetic declination. The gravitation force components (*g_x_*,*g_y_*,*g_z_*) are determined from Equation (3), that is:

(12) $$\begin{array}{l} g_{x}=a_{x}-\left[\dot{U}+(\mathrm{Wq}-\mathrm{Vr})\right]\\ g_{y}=a_{y}-\left[\dot{V}+(\mathrm{Ur}-\mathrm{Wp})\right]\\ g_{z}=a_{z}-\left[\dot{W}+(\mathrm{Vp}-\mathrm{Uq})\right] \end{array}$$

Except the measurement of accelerations (*a_x_*,*a_y_*,*a_z_*) and body rates (*p*,*q*,*r*), the velocities (*U*,*V*,*W*) and velocities’ rates (*U̇*, *V̇*, *Ẇ*) along the body axes are also required. This information can be determined from:

(13) $$\begin{array}{l} U=V_{T}\;{\left(1+{\text{tan}}^{2}\;\alpha +{\text{tan}}^{2}\;\beta \right)}^{\frac{-1}{2}}\\ V=V_{T}\;{\left(1+{\text{tan}}^{2}\;\alpha +{\text{tan}}^{2}\;\beta \right)}^{\frac{-1}{2}}\;\text{tan}\;\beta \\ W=V_{T}\;{\left(1+{\text{tan}}^{2}\;\alpha +{\text{tan}}^{2}\;\beta \right)}^{\frac{-1}{2}}\;\text{tan}\;\alpha \end{array}$$

where *V_T_* is the airspeed, *α* is the angle of attack, and α is the side slip angle. This approach requires an angle of attack sensor and a sideslip sensor to acquire the angle of attack and angle of sideslip information. Equation (13) is valid only for no wind is present. Therefore, in this study, the inertial velocity components (*U*,*V*,*W*) are determined from the ground speed information (*V_N_*, the north velocity, *V_E_*, the east velocity, and *V_D_*, the down velocity of the aircraft) from the GPS receiver through yaw, pitch, and roll rotation sequence. Equation (14) expresses the coordinate transformation for (*V_N_*, *V_E_*, *V_D_*) and (*U*,*V*,*W*):

(14) $$\begin{array}{lll} \left[\begin{array}{c} U\\ V\\ W \end{array}\right] & = & \left[\begin{array}{ccc} 1 & 0 & 0\\ 0 & \text{cos}\;\phi & \text{sin}\;\phi \\ 0 & -\text{sin}\;\phi & \text{cos}\;\phi \end{array}\right]\;\left[\begin{array}{ccc} \text{cos}\;\theta & 0 & -\text{sin}\;\theta \\ 0 & 1 & 0\\ \text{sin}\;\theta & 0 & \text{cos}\;\theta \end{array}\right]\;\left[\begin{array}{ccc} \text{cos}\;\psi & \text{sin}\;\psi & 0\\ -\text{sin}\;\psi & \text{cos}\;\psi & 0\\ 0 & 0 & 1 \end{array}\right]\;\left[\begin{array}{c} V_{N}\\ V_{E}\\ V_{D} \end{array}\right]\\ & = & \left[\begin{array}{ccc} \text{cos}\;\psi \;\text{cos}\;\theta & \text{sin}\;\psi \;\text{cos}\;\theta & -\text{sin}\;\theta \\ -\text{sin}\;\psi \;\text{cos}\;\phi +\text{cos}\;\psi \;\text{sin}\;\theta \;\text{sin}\;\phi & \text{cos}\;\psi \;\text{cos}\;\phi +\text{sin}\;\psi \;\text{sin}\;\theta \;\text{sin}\;\phi & \text{cos}\;\theta \;\text{sin}\;\phi \\ \text{sin}\;\psi \;\text{sin}\;\phi +\text{cos}\;\psi \;\text{sin}\;\theta \;\text{cos}\;\phi & -\text{cos}\;\psi \;\text{sin}\;\phi +\text{sin}\;\psi \;\text{sin}\;\theta \;\text{cos}\;\phi & \text{cos}\;\theta \;\text{cos}\;\phi \end{array}\right]\;\left[\begin{array}{c} V_{N}\\ V_{E}\\ V_{D} \end{array}\right] \end{array}$$

The velocities’ rates (*U̇*, *V̇*, *Ẇ*) are computed from (*V̇_N_*, *V̇_E_*, *V̇_D_*) through the coordinate transformation as in Equation (14) for (*U*,*V*,*W*). The velocities’ rates (*V̇_N_*, *V̇_E_*, *V̇_D_*) are computed from (*V_N_*, *V_E_*, *V_D_*) by the linear approximation *V̇_N_* = [*V_N_*(*n*) – *V_N_*(*n* – 1)] / Δ*t*, *V̇_E_* = [*V_E_*(*n*) – *V_E_*(*n* – 1)] / Δ*t*, and *V̇_D_* = [*V_D_*(*n*) – *V_D_*(*n* – 1)] / Δ*t*. The angle of attack and sideslip sensors are not installed on the airplane when conducting the flight test for this research. Therefore, the results of using Equation (13) to compute the inertial velocity components are not investigated in this paper.

**Figure 6.**
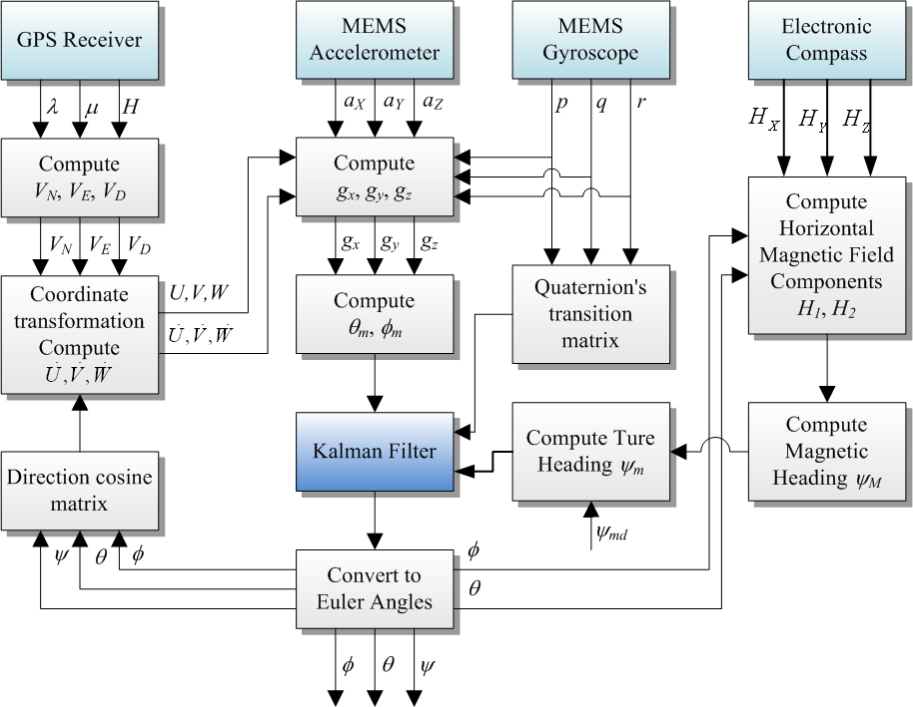
Computation process for attitude determination.

**Figure 7.**
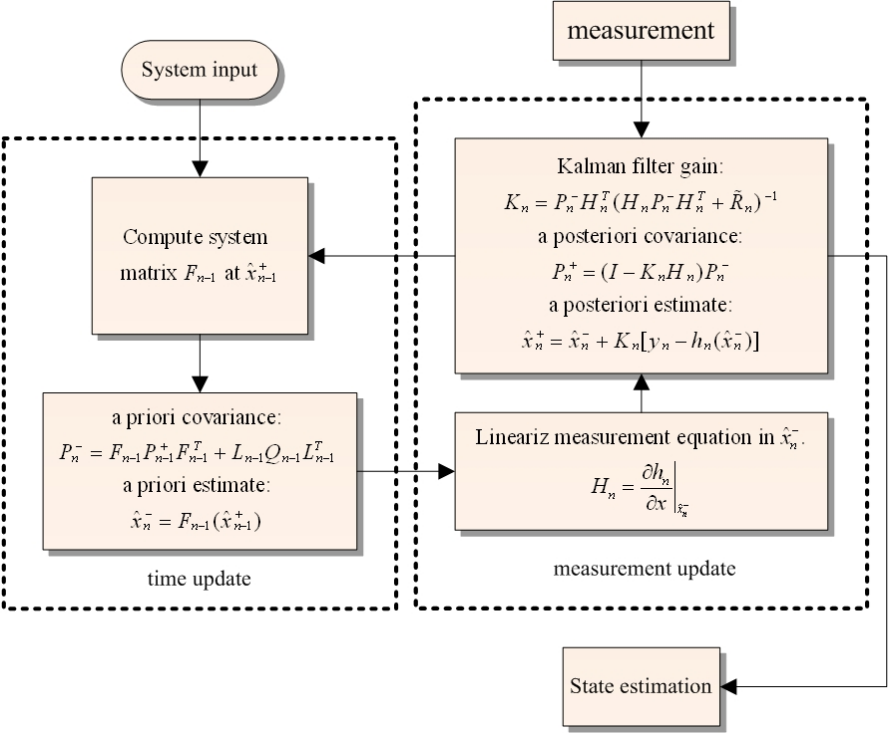
Extended Kalman Filter.

For real time computation, the dynamical system in Equation (10) is expressed in discrete time representation as:

(15) $$\begin{array}{c} x(n+1)=F(n)x(n)+w(n)\\ y(n)=h[x(n)]+v(n) \end{array}$$

where *F*(*n*) = *e^A^*^Δ^*^t^*, which can be represented by the following form [17]:

(16) $$\begin{array}{c} F(n)=\left[\begin{array}{cccc} \text{cos}\left(\frac{\left\|\vartheta \right\|}{2}\right) & -\frac{\Delta P(n)}{\left\|\vartheta \right\|}\;\text{sin}\;\left(\frac{\left\|\vartheta \right\|}{2}\right) & -\frac{\Delta Q(n)}{\left\|\vartheta \right\|}\;\text{sin}\;\left(\frac{\left\|\vartheta \right\|}{2}\right) & -\frac{\Delta R(n)}{\left\|\vartheta \right\|}\;\text{sin}\;\left(\frac{\left\|\vartheta \right\|}{2}\right)\\ \frac{\Delta P(n)}{\left\|\vartheta \right\|}\;\text{sin}\;\left(\frac{\left\|\vartheta \right\|}{2}\right) & \text{cos}\;\left(\frac{\left\|\vartheta \right\|}{2}\right) & \frac{\Delta R(n)}{\left\|\vartheta \right\|}\;\text{sin}\;\left(\frac{\left\|\vartheta \right\|}{2}\right) & -\frac{\Delta Q(n)}{\left\|\vartheta \right\|}\;\text{sin}\;\left(\frac{\left\|\vartheta \right\|}{2}\right)\\ \frac{\Delta Q(n)}{\left\|\vartheta \right\|}\;\text{sin}\;\left(\frac{\left\|\vartheta \right\|}{2}\right) & -\frac{\Delta R(n)}{\left\|\vartheta \right\|}\;\text{sin}\;\left(\frac{\left\|\vartheta \right\|}{2}\right) & \text{cos}\;\left(\frac{\left\|\vartheta \right\|}{2}\right) & \frac{\Delta P(n)}{\left\|\vartheta \right\|}\;\text{sin}\;\left(\frac{\left\|\vartheta \right\|}{2}\right)\\ \frac{\Delta R(n)}{\left\|\vartheta \right\|}\;\text{sin}\;\left(\frac{\left\|\vartheta \right\|}{2}\right) & \frac{\Delta Q(n)}{\left\|\vartheta \right\|}\;\text{sin}\;\left(\frac{\left\|\vartheta \right\|}{2}\right) & -\frac{\Delta P(n)}{\left\|\vartheta \right\|}\;\text{sin}\;\left(\frac{\left\|\vartheta \right\|}{2}\right) & \text{cos}\;\left(\frac{\left\|\vartheta \right\|}{2}\right) \end{array}\right]\\ \vartheta =\left[\begin{array}{ccc} \Delta P & \Delta Q & \Delta R \end{array}\right]\;,\;\left\|\vartheta \right\|=\sqrt{{\left(\Delta P\right)}^{2}+{\left(\Delta Q\right)}^{2}+{\left(\Delta R\right)}^{2}} \end{array}$$

with Δ*P*(*n*) = *p*(*n*)Δ*t*, Δ*Q*(*n*) = *q*(*n*)Δ*t*, Δ*R*(*n*) = *r*(*n*)Δ*t*, Δ*t* is the sampling time (0.05 s in this study), *p*(*n*), *q*(*n*), *r*(*n*) are the pitch rate, roll rate, and yaw rate at time index *n*. The output function—*h*[*x*(*n*)]—is nonlinear. Therefore, the extended Kalman filter is chosen to perform the filtering for the attitude determination in this study. The computation process is depicted in Figure 6, and the Kalman filtering details are shown in Figure 7. In the estimation process, after initialization, the iteration loop starts with computing the process matrix *F*(*n*−1) and the process noise variance, after which the time updates of the state estimate ${\hat{x}}_{n}^{-}$ and estimation-error covariance $P_{n}^{-}$ are performed. Subsequently, the linearized measurement function *H_n_* and the variances of the measurement noises are determined. The function *H_n_* is obtained by taking the derivatives of the observation function *h*(*x*) with respect to the state of the filter. The state of the filter is the quaternion components. Therefore, the function *H_n_* is given in the following form:

(17) $$H_{n}={\frac{\partial h}{\partial x}|}_{{\hat{x}}^{-}}=\left[\begin{array}{cccc} \frac{\partial \phi }{\partial e_{0}} & \frac{\partial \phi }{\partial e_{1}} & \frac{\partial \phi }{\partial e_{2}} & \frac{\partial \phi }{\partial e_{3}}\\ \frac{\partial \theta }{\partial e_{0}} & \frac{\partial \theta }{\partial e_{1}} & \frac{\partial \theta }{\partial e_{2}} & \frac{\partial \theta }{\partial e_{3}}\\ \frac{\partial \psi }{\partial e_{0}} & \frac{\partial \psi }{\partial e_{1}} & \frac{\partial \psi }{\partial e_{2}} & \frac{\partial \psi }{\partial e_{3}}\\ 2e_{0} & 2e_{1} & 2e_{2} & 2e_{3} \end{array}\right]$$

With all the necessary information available, the last step of the iteration loop is to perform the measurement update of the state estimate ${\hat{x}}_{n}^{+}$ and estimation-error covariance $P_{n}^{+}$. Details of the process and measurement noises are presented in the following section.

## 5. Noise Characteristics

In the Kalman filter design, we use the covariances of the process noise and measurement noise as design parameters to achieve minimum variance of the estimation error. Hence, we must determine the variances of the process and measurement noises as precisely as possible. For the attitude determination considered in this study, the process noises are mainly derived from the outputs of the gyros (*p*,*q*,*r*). Assuming that that *p* = *p̄* + *p̃*, *q* = *q̄* + *q̃*, and *r* = *r̄* + *r̃*, with *p̄*, *q̄*, and *r̄* the mean of *p*, *q*, and *r* respectively, and *p̃*, *q̃*, and *r̃* are the deviations of *p*, *q*, and *r*, Equation (9) can be written as:

(18) $$\begin{array}{lll} \left[\begin{array}{c} {\dot{e}}_{0}\\ {\dot{e}}_{1}\\ {\dot{e}}_{2}\\ {\dot{e}}_{3} \end{array}\right] & = & \;\frac{1}{2}\cdot \left[\begin{array}{cccc} 0 & -(\bar{p}+\tilde{p}) & -(\bar{q}+\tilde{q}) & -(\bar{r}+\tilde{r})\\ \left(\bar{p}+\tilde{p}\right) & 0 & \left(\bar{r}+\tilde{r}\right) & -(\bar{q}+\tilde{q})\\ \left(\bar{q}+\tilde{q}\right) & -(\bar{r}+\tilde{r}) & 0 & \left(\bar{p}+\tilde{p}\right)\\ \left(\bar{r}+\tilde{r}\right) & \left(\bar{q}+\tilde{q}\right) & -(\bar{p}+\tilde{p}) & 0 \end{array}\right]\;\left[\begin{array}{c} e_{0}\\ e_{1}\\ e_{2}\\ e_{3} \end{array}\right]\\ & = & \frac{1}{2}\cdot \left[\begin{array}{cccc} 0 & -\bar{p} & -\bar{q} & -\bar{r}\\ \bar{p} & 0 & \bar{r} & -\bar{q}\\ \bar{q} & -\bar{r} & 0 & \bar{p}\\ \bar{r} & \bar{q} & -\bar{p} & 0 \end{array}\right]\;\left[\begin{array}{c} e_{0}\\ e_{1}\\ e_{2}\\ e_{3} \end{array}\right]+\frac{1}{2}\;\left[\begin{array}{ccc} -e_{1} & -e_{2} & -e_{3}\\ e_{0} & -e_{3} & e_{2}\\ e_{3} & e_{0} & -e_{1}\\ -e_{2} & e_{1} & e_{0} \end{array}\right]\;\left[\begin{array}{c} \tilde{p}(t)\\ \tilde{q}(t)\\ \tilde{r}(t) \end{array}\right] \end{array}$$

The second term on the right hand side is considered as the process noise *w*(*t*) in this study. In the sequel, *φ̄* represents the expectation or mean of the signal *φ*, and *φ̃* indicates the deviation from the mean value.

For a discrete time system, the process noise *w*(*n*) is:

(19) $$w(n)=\frac{1}{2}\;\left[\begin{array}{ccc} -e_{1} & -e_{2} & -e_{3}\\ e_{0} & -e_{3} & e_{2}\\ e_{3} & e_{0} & -e_{1}\\ -e_{2} & e_{1} & e_{0} \end{array}\right]\;\left[\begin{array}{c} \tilde{p}(n)\\ \tilde{q}(n)\\ \tilde{r}(n) \end{array}\right]$$

It is simple to show that the variance of the process noise is $L_{n-1}\;Q_{n-1}\;L_{n-1}^{T}$ with:

(20) $$L_{n-1}=\frac{1}{2}\;\left[\begin{array}{ccc} -e_{1} & -e_{2} & -e_{3}\\ e_{0} & -e_{3} & e_{2}\\ e_{3} & e_{0} & -e_{1}\\ -e_{2} & e_{1} & e_{0} \end{array}\right]\;\;\;,\;\;\;Q_{n-1}=\left[\begin{array}{ccc} \sigma_{p}^{2} & 0 & 0\\ 0 & \sigma_{q}^{2} & 0\\ 0 & 0 & \sigma_{r}^{2} \end{array}\right]$$

where $\sigma_{p}^{2}$, $\sigma_{q}^{2}$, and $\sigma_{r}^{2}$ are the variances of *p*, *q*, and *r*, respectively. The characteristics of the sources of the measurement determine the variances of the measurement noises. In this study, the pitch angle *θ* and roll angle *ϕ* are computed from the measured gravity components in Equations (1) and (12), while the heading angle is determined from the electronic compass system in Equation (6). We can therefore represent the variance of the measurement noises in the following form:

(21) $$\tilde{R}=\left[\begin{array}{ccc} \sigma_{\theta \phi }^{2} & 0 & 0\\ 0 & \sigma_{\psi }^{2} & 0\\ 0 & 0 & \sigma_{C}^{2} \end{array}\right]$$

where $\sigma_{\theta \phi }^{2}$ represents the variance of pitch and roll angles (*θ*,*ϕ*); $\sigma_{\psi }^{2}$ is the variance of heading angle; $\sigma_{C}^{2}$ is the variance of the constraint in Equation (8), and is equal to zero. To avoid numerical problem, the variance $\sigma_{C}^{2}$ can be set to a small constant instead of zero. Since the pitch and roll angles are computed from Equation (12), the deviations of *g_x_*, *g_y_*, and *g_z_*, denoted as g̃*_x_*, g̃*_y_*, and g̃*_z_*, can be decided by expanding Equation (12) in a Taylor series about the mean of the measured variables, (*a_x_*,*a_y_*,*a_z_*), (*U*,*V*,*W*), (*U̇*, *V̇*, *Ẇ*), (*p*,*q*,*r*) and neglecting the second order terms to obtain:

(22) $$\left[\begin{array}{c} {\tilde{g}}_{x}\\ {\tilde{g}}_{y}\\ {\tilde{g}}_{z} \end{array}\right]=M_{\mathrm{xyz}}\;\xi$$

where:

(23) $$\begin{array}{lll} M_{\mathrm{xyz}} & = & \left[\begin{array}{cccccccccccc} 1 & 0 & 0 & -1 & 0 & 0 & 0 & r & -q & 0 & -W & V\\ 0 & 1 & 0 & 0 & -1 & 0 & -r & 0 & p & W & 0 & -U\\ 0 & 0 & 1 & 0 & 0 & -1 & q & -p & 0 & -V & U & 0 \end{array}\right]\\ \xi & = & {\left[\begin{array}{llllllllllll} {\tilde{a}}_{x} & {\tilde{a}}_{y} & {\tilde{a}}_{z} & \tilde{\dot{U}} & \tilde{\dot{V}} & \tilde{\dot{W}} & \tilde{U} & \tilde{V} & \tilde{W} & \tilde{p} & \tilde{q} & \tilde{r} \end{array}\right]}^{T} \end{array}$$

The roll angle *ϕ* and pitch angle *θ* are computed from:

(24) $$\phi ={\text{tan}}^{-1}\left(\frac{g_{y}}{g_{z}}\right)\;\;\;,\;\;\;\theta ={\text{tan}}^{-1}\left(\frac{-g_{x}\;\text{cos}\;\phi }{g_{z}}\right)$$

Expanding the function $\left(\frac{g_{y}}{g_{z}}\right)$ in the Taylor series about the mean values and neglecting the second and higher order terms, the deviation of $\left(\frac{g_{y}}{g_{z}}\right)$ can be approximated by:

(25) $$\tilde{\left(\frac{g_{y}}{g_{z}}\right)}=\left[\begin{array}{lll} 0 & \frac{1}{g_{z}} & -\frac{g_{y}}{g_{z}^{2}} \end{array}\right]\;\left[\begin{array}{l} {\tilde{g}}_{x}\\ {\tilde{g}}_{y}\\ {\tilde{g}}_{z} \end{array}\right]$$

To determine *ϕ̃*, the function $\phi ={\text{tan}}^{-1}\;\left(\frac{g_{y}}{g_{z}}\right)$ is also expanded in a Taylor series, and the deviation *ϕ̃* is approximated as:

(26) $$\tilde{\phi }=\frac{1}{1+{\left(\frac{g_{y}}{g_{z}}\right)}^{2}}\;\tilde{\left(\frac{g_{y}}{g_{z}}\right)}=\left[\begin{array}{lll} 0 & \frac{g_{z}}{g_{y}^{2}+g_{z}^{2}} & -\frac{g_{y}}{g_{y}^{2}+g_{z}^{2}} \end{array}\right]\;\left[\begin{array}{l} {\tilde{g}}_{x}\\ {\tilde{g}}_{y}\\ {\tilde{g}}_{z} \end{array}\right]$$

Similarly, the deviation of $\left(\frac{-g_{x}\;\text{cos}\;\phi }{g_{z}}\right)$ can be approximated by:

(27) $$\begin{array}{lll} \left(\frac{-\tilde{g_{x}\;\text{cos}}\;\phi }{g_{z}}\right) & = & -\frac{\text{cos}\;\phi }{g_{z}}\;{\tilde{g}}_{x}+\frac{g_{x}\;\text{cos}\;\phi }{g_{z}^{2}}\;{\tilde{g}}_{z}+\frac{g_{x}\;\text{sin}\;\phi }{g_{z}}\;\tilde{\phi }\\ & = & -\frac{\text{cos}\;\phi }{g_{z}}\;{\tilde{g}}_{x}+\frac{g_{x}\;\text{cos}\;\phi }{g_{z}^{2}}\;{\tilde{g}}_{z}+\frac{g_{x}\;\text{sin}\;\phi }{g_{z}}\;\left[\begin{array}{lll} 0 & \frac{g_{z}}{g_{y}^{2}+g_{z}^{2}} & -\frac{g_{y}}{g_{y}^{2}+g_{z}^{2}} \end{array}\right]\;\left[\begin{array}{l} {\tilde{g}}_{x}\\ {\tilde{g}}_{y}\\ {\tilde{g}}_{z} \end{array}\right]\\ & = & \left[\begin{array}{ccc} -\frac{\text{cos}\;\phi }{g_{z}} & \frac{g_{x}\;\text{sin}\;\phi }{g_{y}^{2}+g_{z}^{2}} & \frac{g_{x}\;\text{cos}\;\phi }{g_{z}^{2}}-\frac{g_{x}g_{y}\;\text{sin}\;\phi }{g_{z}\;(g_{y}^{2}+g_{z}^{2})} \end{array}\right]\;\left[\begin{array}{l} {\tilde{g}}_{x}\\ {\tilde{g}}_{y}\\ {\tilde{g}}_{z} \end{array}\right] \end{array}$$

Thus, the deviation of $\theta ={\text{tan}}^{-1}\left(\frac{-g_{x}\;\text{cos}\;\phi }{g_{z}}\right)$, after a Taylor series expansion and neglecting the higher order terms, can be obtained as:

(28) $$\begin{array}{lll} \tilde{\theta } & = & \frac{1}{1+{\left(\frac{-g_{x}\;\text{cos}\;\phi }{g_{x}}\right)}^{2}}\;\left[\begin{array}{ccc} -\frac{\text{cos}\;\phi }{g_{z}} & \frac{g_{x}\;\text{sin}\;\phi }{g_{y}^{2}+g_{z}^{2}} & \frac{g_{x}\;\text{cos}\;\phi }{g_{z}^{2}}-\frac{g_{x}g_{y}\;\text{sin}\;\phi }{g_{z}(g_{y}^{2}+g_{z}^{2})} \end{array}\right]\;\left[\begin{array}{l} {\tilde{g}}_{x}\\ {\tilde{g}}_{y}\\ {\tilde{g}}_{z} \end{array}\right]\\ & = & \left[\begin{array}{ccc} \frac{-g_{z}\;\text{cos}\;\phi }{g_{x}^{2}\;{\text{cos}}^{2}\;\phi +g_{z}^{2}} & \frac{g_{x}g_{z}^{2}\;\text{sin}\;\phi }{\left(g_{y}^{2}+g_{z}^{2})(g_{x}^{2}\;{\text{cos}}^{2}\;\phi +g_{z}^{2}\right)} & \frac{(g_{y}^{2}+g_{z}^{2})g_{x}\;\text{cos}\;\phi -g_{x}\;g_{y}\;g_{z}\;\text{sin}\;\phi }{\left(g_{y}^{2}+g_{z}^{2})(g_{x}^{2}\;{\text{cos}}^{2}\;\phi +g_{z}^{2}\right)} \end{array}\right]\;\left[\begin{array}{c} {\tilde{g}}_{x}\\ {\tilde{g}}_{y}\\ {\tilde{g}}_{z} \end{array}\right] \end{array}$$

Defining:

(29) $$M_{\phi \theta }=\left[\begin{array}{ccc} 0 & \frac{g_{z}}{g_{y}^{2}+g_{z}^{2}} & -\frac{g_{y}}{g_{y}^{2}+g_{z}^{2}}\\ \frac{-g_{z}\;\text{cos}\;\phi }{g_{x}^{2}\;{\text{cos}}^{2}\;\phi +g_{z}^{2}} & \frac{g_{x}\;g_{z}^{2}\;\text{sin}\;\phi }{\left(g_{y}^{2}+g_{z}^{2})(g_{x}^{2}\;{\text{cos}}^{2}\;\phi +g_{z}^{2}\right)} & \frac{(g_{y}^{2}+g_{z}^{2})g_{x}\;\text{cos}\;\phi -g_{x}\;g_{y}\;g_{z}\;\text{sin}\;\phi }{\left(g_{y}^{2}+g_{z}^{2})(g_{x}^{2}\;{\text{cos}}^{2}\;\phi +g_{z}^{2}\right)} \end{array}\right]$$

Then:

(30) $$\left[\begin{array}{c} \tilde{\phi }\\ \tilde{\theta } \end{array}\right]=M_{\phi \theta }\;\left[\begin{array}{c} {\tilde{g}}_{x}\\ {\tilde{g}}_{y}\\ {\tilde{g}}_{z} \end{array}\right]=M_{\phi \theta }\;M_{\mathrm{xyz}}\;\xi$$

Thus, the variance of pitch and roll angles, $\sigma_{\theta \phi }^{2}$, is:

(31) $$\begin{array}{lll} \sigma_{\theta \phi }^{2} & = & E\;\left[\left(M_{\phi \theta }\;M_{\mathrm{xyz}}\;\xi \right)\;{\left(M_{\phi \theta }\;M_{\mathrm{xyz}}\;\xi \right)}^{T}\right]=M_{\phi \theta }\;M_{\mathrm{xyz}}\;E\;\left[{\xi \xi }^{T}\right]\;M_{\mathrm{xyz}}^{T}\;M_{\phi \theta }^{T}\\ & = & M_{\phi \theta }\;M_{\mathrm{xyz}}\;R_{\phi \theta }\;M_{\mathrm{xyz}}^{T}\;M_{\phi \theta }^{T} \end{array}$$

where:

(32) $$R_{\phi \theta }=\mathrm{diag}\;\left[\begin{array}{llllllllllll} \sigma_{a_{X}}^{2} & \sigma_{a_{Y}}^{2} & \sigma_{a_{Z}}^{2} & \sigma_{\dot{U}}^{2} & \sigma_{\dot{V}}^{2} & \sigma_{\dot{W}}^{2} & \sigma_{U}^{2} & \sigma_{V}^{2} & \sigma_{W}^{2} & \sigma_{p}^{2} & \sigma_{q}^{2} & \sigma_{r}^{2} \end{array}\right]$$

and $\sigma_{\psi }^{2}$ represents the variance of the signal *φ*. Significantly, in Equation (32) all noise sources are assumed to be uncorrelated.

The variance of the heading angle $\sigma_{\psi }^{2}$ can be obtained in a similar manner. To determine the heading angle, *H*_1_ and *H*_2_ are computed based on the filtered roll and pitch angles. Thus, the deviations of *H̃*_1_ and *H̃*_2_ can be expressed as:

(33) $$\begin{array}{lll} \left[\begin{array}{c} {\tilde{H}}_{1}\\ {\tilde{H}}_{2} \end{array}\right] & = & M_{H12}\;\left[\begin{array}{c} {\tilde{H}}_{X}\\ {\tilde{H}}_{Y}\\ {\tilde{H}}_{Z} \end{array}\right]\\ M_{H12} & = & \left[\begin{array}{ccc} \text{cos}\;\theta & \text{sin}\;\theta \;\text{sin}\;\phi & \text{cos}\;\phi \;\text{sin}\;\theta \\ 0 & \text{cos}\;\phi & -\text{sin}\;\phi \end{array}\right] \end{array}$$

Hence the deviation of $\left(\frac{H_{1}}{H_{2}}\right)$ can be approximated by:

(34) $$\tilde{\left(\frac{H_{2}}{H_{1}}\right)}=\frac{1}{H_{1}}\;{\tilde{H}}_{2}-\frac{H_{2}}{H_{1}^{2}}\;{\tilde{H}}_{1}=\left[\begin{array}{cc} -\frac{H_{2}}{H_{1}^{2}} & \frac{1}{H_{1}} \end{array}\right]\;\left[\begin{array}{c} {\tilde{H}}_{1}\\ {\tilde{H}}_{2} \end{array}\right]$$

Therefore, the deviation of the magnetic heading $\psi_{M}={\text{tan}}^{-1}\left(\frac{H_{2}}{H_{1}}\right)$ after a Taylor series expansion can be approximated by:

(35) $$\begin{array}{lll} {\tilde{\psi }}_{M} & = & \frac{1}{1+{\left(\frac{H_{2}}{H_{1}}\right)}^{2}}\tilde{\left(\frac{H_{2}}{H_{1}}\right)}=\frac{H_{1}^{2}}{H_{1}^{2}+H_{2}^{2}}\;\left[\begin{array}{cc} -\frac{H_{2}}{H_{1}^{2}} & \frac{1}{H_{1}} \end{array}\right]\;\left[\begin{array}{c} {\tilde{H}}_{1}\\ {\tilde{H}}_{2} \end{array}\right]=M_{\psi }\;\left[\begin{array}{c} {\tilde{H}}_{1}\\ {\tilde{H}}_{2} \end{array}\right]\\ M_{\psi } & = & \left[\begin{array}{cc} -\frac{H_{2}}{H_{1}^{2}+H_{2}^{2}} & \frac{H_{1}}{H_{1}^{2}+H_{2}^{2}} \end{array}\right] \end{array}$$

Thus, the variance of the heading angle $\sigma_{\psi }^{2}$ is:

(36) $$\begin{array}{lll} \sigma_{\psi M}^{2} & = & E\;\left\{\left(M_{\psi }\;M_{H12}\;\left[\begin{array}{l} {\tilde{H}}_{X}\\ {\tilde{H}}_{Y}\\ {\tilde{H}}_{Z} \end{array}\right]\right)\;{\left(M_{\psi }\;M_{H12}\;\left[\begin{array}{l} {\tilde{H}}_{X}\\ {\tilde{H}}_{Y}\\ {\tilde{H}}_{Z} \end{array}\right]\right)}^{T}\right\}\\ & = & M_{\psi }\;M_{H12}\;R_{\psi }\;M_{H12}^{T}\;M_{\psi }^{T} \end{array}$$

where $R_{\psi }=\mathrm{diag}\;[\sigma_{H_{X}}^{2}\;\sigma_{H_{Y}}^{2}\;\sigma_{H_{Z}}^{2}]$, $\sigma_{H_{X}}^{2}$, $\sigma_{H_{Y}}^{2}$, and $\sigma_{H_{Z}}^{2}$ are the variances of *H_X_*, *H_Y_*, and *H_Z_*, respectively. We also assume that *H̃_X_*, *H̃_Y_*, and *H̃_Z_* are all uncorrelated. The variance of the heading angle $\sigma_{\psi }^{2}$ is equal to $\sigma_{\psi_{M}}^{2}$ since the only difference between the heading angle *ψ* and the magnetic heading *ψ_M_* is the magnetic declination angle.

## 6. Design Evaluation

**Figure 8.**
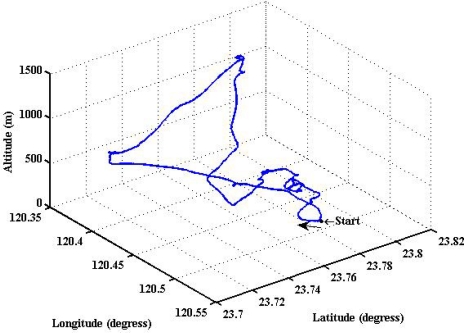
Trajectory of the complete flight.

**Figure 9.**
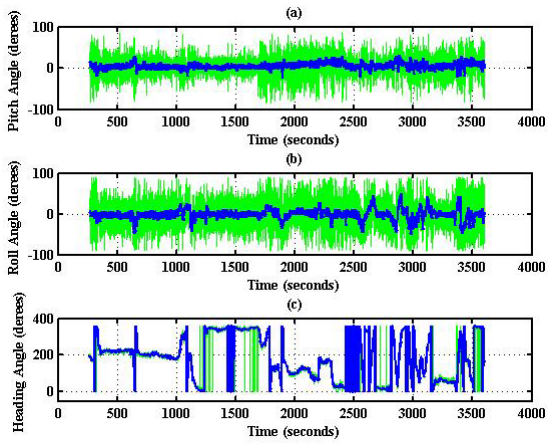
Evolutions of the attitude angles.

To evaluate the design of the flight information measurement unit and the extended Kalman filtering for attitude determination, the system is tested on an ultralight aircraft. The test data—including the three axes body rates (*p*,*q*,*r*), accelerations (*a_x_*,*a_y_*,*a_z_*), magnetic field (*H_X_*,*H_Y_*,*H_Z_*), and information from GPS receiver—are recorded during flight test. The flight was performed in Central Taiwan at approximately 120.44 degrees East Longitude and 23.77 degrees North Latitude area. The flight trajectory is shown in Figure 8. The evolutions of the attitude, including pitch, roll, and heading angles are shown in Figure 9. The attitude angles (directly generated from the measured data and computed after the Kalman filtering) are both shown in Figure 9. The duration of the flight covers a total of 3,620 s. The results are further examined and discussed below. In particular, during certain time intervals, the aircraft performed specific flight trajectories: during the time interval 1,106–1,130 s, the aircraft was making a left turn; from 1,420–1,430 s, the aircraft ascended; the aircraft descended during the time interval 2,340–2,380 s; during the time interval 2,600–2,675 s, the aircraft was making a right turn; from 2,745–2,755 s, the aircraft was in level flight with wing wiggling; and from 2,810–2,900 s, the aircraft was in S-turn maneuver. Much of the noisy signals of the attitude angles in the followings figures are the results deduced from the measurement, whereas the less noisy signals are the results of the filtered signals.

**Figure 10.**
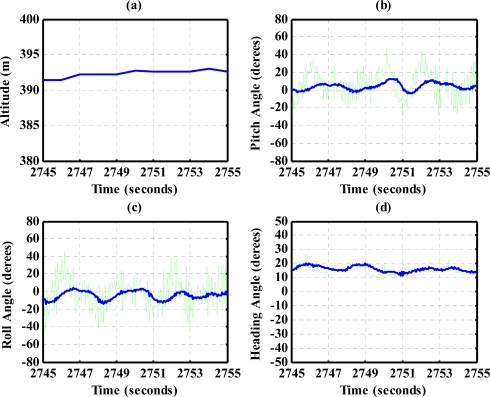
Nearly level flight with wing wiggling.

**Figure 11.**
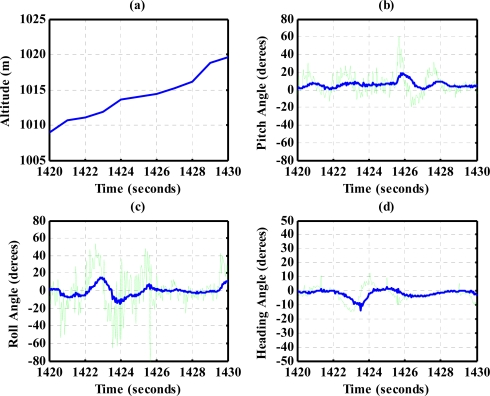
Attitude for climbing-up condition.

**Figure 12.**
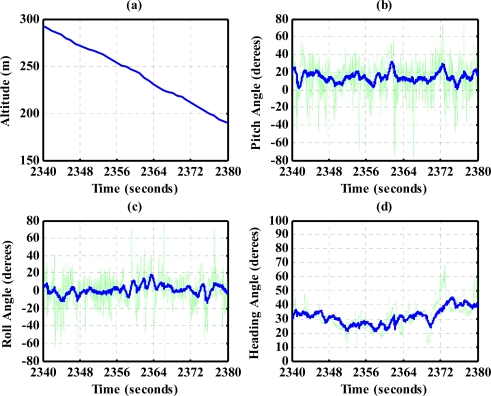
Attitude during the descent flight.

**Figure 13.**
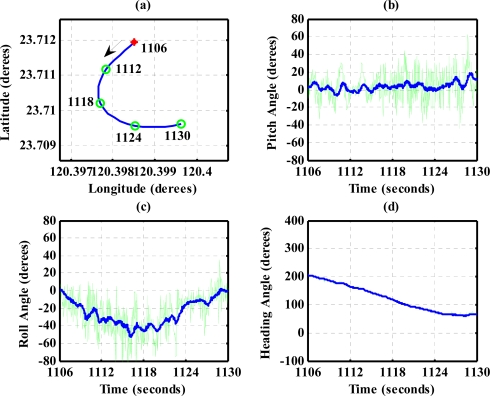
Attitude evolutions during the time period 1,106–1,130 s (making a left turn). The arrow in (a) shows the direction of flight.

**Figure 14.**
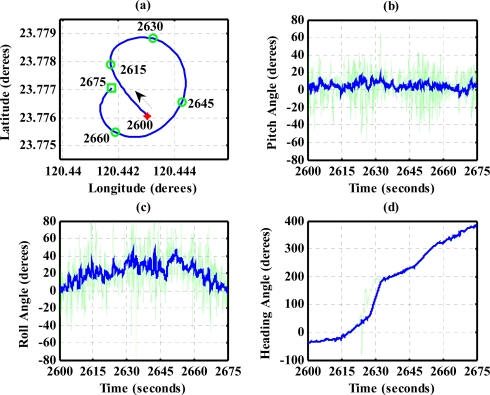
Attitude evolutions during the time period 2,600–2,675 s (making a right turn). The arrow in (a) shows the direction of flight.

**Figure 15.**
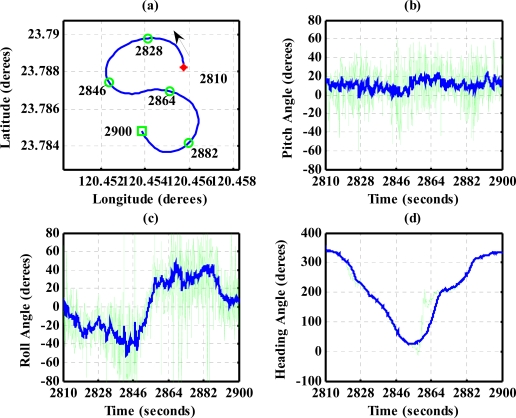
Attitude evolutions during an S-turn maneuver. The arrow in (a) shows the direction of flight.

The results for nearly level flight with wing wiggling (intentionally performed) are shown in Figure 10. In this phase, the altitude was sustained at approximately 392 to 393 meters height, as shown in Figure 10(a). The roll angle, as shown in Figure 10(c), responded as commanded. Figure 10(d) shows the tendency of turning to the left. The average of the roll angles in Figure 10(c) is negative, which confirms that the airplane is turning left slowly. The results also show that the time delay of the attitude estimation is approximately 0.5 s due to filter computation. Figure 11 indicates the results for the climbing up condition. During the time period of 1,420 to 1,430 s, the airplane climbed from 1,009 m to 1,019 m height. The results during the descent flight (time duration 2,340 to 2,380 s) are shown in Figure 12. In this descent flight phase, the altitude decreases from 290 to 190 m height. Evolutions of the pitch angle in Figure 12(b) are all positive, with an average of roughly 10 degrees (which is normal for aircraft descent flight). Figure 13 shows the results when the airplane made a left turn. The data from the GPS receiver, as shown in Figure 13(a), indicate that the airplane is turning left. When making a left turn, the airplane banks to the left before starting to turn. The results in Figure 13(c), negative roll angles, show that the airplane rolls to the left, which confirms that the airplane is turning to the left. The heading angle changes from approximately 200 degrees to 60 degrees in Figure 13(d), which verifies that the airplane is making a left turn. Figure 14 presents the results when the airplane made a right turn. In the right turning phase, the airplane has a positive roll angle, which is evident in Figure 14(c). During the time period from 2,810 to 2,900 s, the airplane made an S-turn. Evolutions of the attitude angles during this maneuver are shown in Figure 15. Figure 15(a) shows the trajectory of the S-turn maneuver from the GPS data. During this particular maneuver, the airplane makes a left turn before following with a right turn. Figure 15(c) shows that the roll angle turns to a negative value first, then returns to zero degrees for the left maneuver. Thereafter, it turns positive before returning to zero degrees for a right turning operation. The heading angle shown in Figure 15(d) starts from approximately 340 degrees and gradually decreases to roughly 25 degrees, then increases back to 335 degrees, which correctly corresponds to the S-turn operation.

From the above examinations, we demonstrated that the proposed attitude determination algorithm estimates the attitude angles successfully. The algorithm is also reliable for long-term and high-dynamic maneuvers.

**Figure 16.**
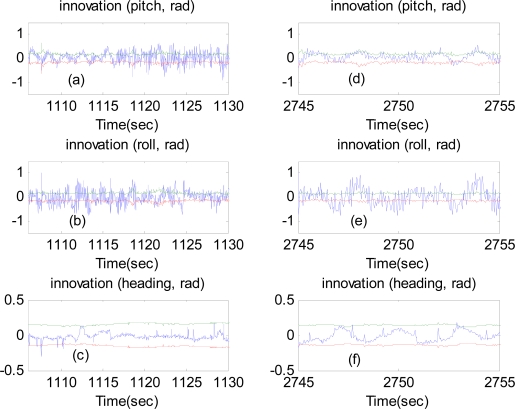
Innovation around ±3 standard deviations. Green and red lines are the boundaries of the ±3 standard deviations.

**Figure 17.**
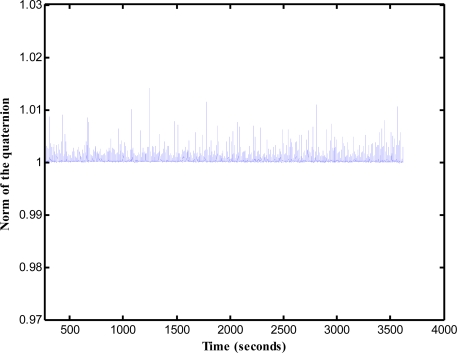
Evolution of the norm of the quaternion vector.

To further investigate the performance of the proposed attitude estimation method, the innovation around ±3 standard deviations (extracted from the innovation covariance) and the quaternion norm constraint in Equation (8) are examined. Figure 16(a–c) represent the results of the innovation when the airplane made a left turn. Figure 16(d–f) are the results for nearly level flight with wing wiggling. At some points, the innovation exceeds the boundaries of the ±3 standard deviations. This is due to the fact that the extended Kalman filter is an approximate solution. It relies on the linearization to propagate the mean and covariance of the state. The norms of the quaternion computed from the extended Kalman filter for all over the duration of flight are displayed in Figure 17. The results show that the constraint optimization is well performed.

**Figure 18.**
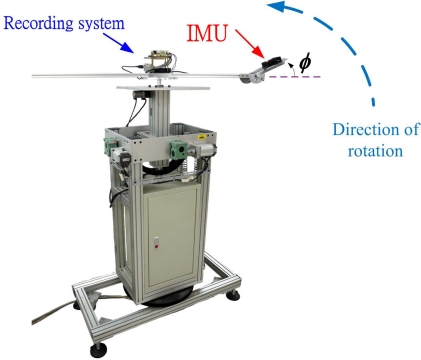
The motion platform and the test arrangement.

Because there is no primary standard, the proposed attitude estimation algorithm is also tested using a motion platform under a controlled environment. An image of the test arrangement is shown in Figure 18. A cantilever beam is mounted atop the motion platform. A specially designed movable mounting plate is constructed at the end of the beam. The IMU is placed on plate. The mounting plate is arranged in such a way that the IMU is mounted with 0 degree pitch angle and −20 degrees roll angles. An on-board computer is mounted on top of the center of the motion platform to record the outputs from the IMU during the test.

**Figure 19.**
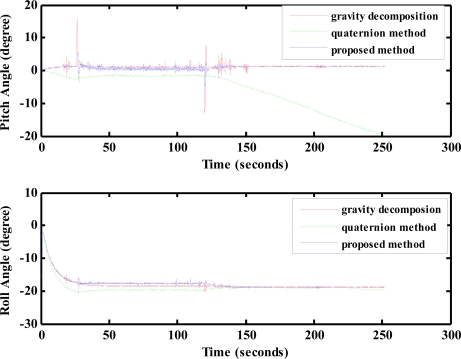
Results from lab test.

The motion platform is controlled to perform uniform circular motion with an angular velocity of 0.873 rad/s. Under this test condition, the velocities (*U*,*V*,*W*) and accelerations (*U̇*, *V̇*, *Ẇ*) along the body axes of the IMU are *U* = 0.4466 m/s, *V* = *W* = 0 m/s, and *U̇* = *V̇* = *Ẇ* 0 m/s^2^. Using these velocity and acceleration information together with the accelerations measured from the accelerometers and the body rates from the gyro outputs, the pitch angle and roll angle are computed by using each of the gravity force components, the Euler quaternion method (which can be considered as a direct 6-DOF mechanization for attitude determination [18]), and the proposed extended Kalman filtering. The results are shown in Figure 19. The results in Figure 19 indicate that the gravity force decomposition will provide accurate results of 0 degree pitch angle and −20 degrees roll angle but suffer from noisy contents. The roll angle is correctly deduced from the quaternion computation with less noisy contents. However, the pitch angle tends to diverge. The results using the proposed extended Kalman filtering provide both accurate and less noisy results for the pitch and roll angles.

## 7. Conclusions

This study presented attitude determination using a low cost MEMS-based flight information measurement unit. The proposed attitude estimation algorithm is intuitive, easy to implement, and reliable for long-term high dynamic maneuvers. The algorithm utilized the evolution of the four elements of the quaternion method as the dynamical model for the Kalman filter. The roll and pitch angles (computed from the gravity force decomposition) and the heading angle (determined from the electronic compass unit) are selected as the measurements of the Kalman filter. The immeasurable gravity accelerations are deduced from the outputs of the three axes accelerometers, the relative accelerations, and the accelerations due to body rotation. The constraint of the four elements of the quaternion method is treated as perfect measurement and is integrated into the filter design. Approximations of the time varying noise variances of the measured signals are established with detailed derivations. The proposed algorithm is successfully demonstrated through a set of flight test data collected from the in house-designed MEMS based attitude determination system. To improve the performance of the attitude estimation, estimation of the gyro drift and velocity derivatives can be included into the Kalman filter formulation with the expense of computation demands.
